# MicroRNA identification using linear dimensionality reduction with explicit feature mapping

**DOI:** 10.1186/1753-6561-7-S7-S8

**Published:** 2013-12-20

**Authors:** Navid Shakiba, Luis Rueda

**Affiliations:** 1 School of Computer Science, University of Windsor 401 Sunset Avenue, Windsor, Ontario, Canada - N9B 3P4

## Abstract

**Background:**

microRNAs are a class of small RNAs, about 20 nt long, which regulate cellular processes in animals and plants. Identifying microRNAs is one of the most important tasks in gene regulation studies. The main features used for identifying these tiny molecules are those in hairpin secondary structures of pre-microRNA.

**Results:**

A new classifier is employed to identify precursor microRNAs from both pseudo hairpins and other non-coding RNAs. This classifier achieves a geometric mean *G_m _*= 92.20% with just three features and 92.91% with seven features.

**Conclusion:**

This study shows that *linear dimensionality reduction *combined with *explicit feature mapping*, namely miLDR-EM, achieves high performance in classification of microRNAs from other sequences. Also, explicitly mapping data onto a high dimensional space could be a useful alternative to kernel-based methods for large datasets with a small number of features. Moreover, we demonstrate that microRNAs can be accurately identified by just using three properties that involve minimum free energy.

## Background

MicroRNAs are a class of small non-coding RNAs that play a crucial role in gene regulation by perfectly or imperfectly binding into three prime untranslated regions $\left(\text{3'}\;\text{UTR}\right)$ in messenger RNAs, and cause repression of translating mRNAs into proteins or their cleavage. Researchers have estimated that about one third of the human genes are regulated by microRNAs [1]. MicroRNAs perform many cellular tasks in cells including controlling cell developmental timing, cell death and stem cell characterization [2]. In addition, many studies show that malfunction of microRNAs may have devastating impacts on cell life and may cause different types of cancer, heart disease and nervous system disorder [1]. Accordingly, identification of microRNA genes is an essential process in discovering microRNA functions and its role in cellular processes.

Earlier, microRNAs were only identified by using experimental methods. Traditional experimental approaches to microRNA discovery include cloning and sequencing [3], and can detect novel microRNAs. Since microRNAs usually express at low levels and depend on tissue and conditions of the cell, these methods may be unable to identify new microRNAs [1]. Recently high-throughput sequencing approaches, in particular, 454 sequencing, have become popular for discovering new microRNAs [4].

Another category of approaches for identifying microRNAs are computational methods. The main idea behind these methods is to analyze hairpin secondary structures of precursor microRNAs (pre-microRNA). At first, non-coding genes are transcribed into primary microRNAs (pri-microRNA) by RNA Polymerase II [5]. Then, the RNase III enzyme, named Drosha and its co-factor, Pasha, processes pri-microRNA to release pre-microRNA, which is about 80 nt long and folds into a hairpin secondary structure. This product is then ready for further processes to change into mature microRNA. Secondary structure of pre-microRNA allows researchers to propose computational methods that can distinguish these sequences from other sequences in the genome.

There are two subcategories for computational methods: comparative and non-comparative ap-proaches. Biologists believe that microRNAs are highly conserved in related genomes [1]. Therefore, some methods use this property of microRNAs and introduce candidate microRNAs which fold into hairpin secondary structure and are conserved in related genomes. The other subcategory of computational methods includes non-comparative methods. These methods are mainly based on analyzing secondary structure of pre-microRNA and are based on extracted precursor micro RNA features, to classify them from other sequences. There are still some major obstacles to overcome. First, there are thousands of other genome sequences which fold into hairpin secondary structure, called "pseudo hairpins" [6]. Second, many other non-coding RNAs such as YRNAs, snRNAs and tRNAs fold into hairpin secondary structure as well. Therefore, the main challenge is to extract features from the sequences in such a way that pre-microRNAs can be distinguished from other non-coding RNAs and pseudo hairpins to classify them effectively.

Previously, many computational methods were proposed for finding novel microRNAs. The first algorithm for finding microRNA genes, miRscan [7], is a comparative method that uses a sliding window to compare and analyze sequences with previously known microRNA sequences. Later, many other comparative and non-comparative methods were proposed - we only mention a few of them here. miRabela [8] uses 40 sequential and structural features to classify microRNAs. Triplet-SVM [9] is a support vector machine (SVM) classifier which uses "contiguous structure-sequence" features to identify microRNAs. Based on Triplet-SVM, miREncoding SVM [10] and MiPred [11] were proposed to improve the performance of Triplet-SVM. miREncoding SVM added 11 global features and performed feature selection to find the best subset of features. MiPred added two thermodynamical features and also replaced the SVM by the random forest learning algorithm. miPred SVM [12] is another microRNA identification system that uses 29 "global and intrinsic folding measures" as features. MicroPred [13] was proposed by Batuwita and Palade and uses 29 features introduced in miPred in addition to 19 newly introduced features. MicroPred uses human pre-microRNAs as the positive class and both genome pseudo hairpins and other non-coding RNAs as the negative class for feeding its SVM. The feature selection method used in [13] is a filter method that does not consider a classifier for selecting a subset of features. Instead, they rely on some discriminant measures before training the SVM-based classifier. Also, Wang *et al. *[14] proposed a feature selection method that is based on GA-SVM. Lately, Xuan *et al. *[15] proposed another pre-microRNA identification method that selects a subset of samples instead of the whole data set for solving the class imbalance problem.

On the other hand, linear dimensionality reduction (LDR) has been shown to be successfully used in pattern recognition and machine learning [16]. However, LDR methods may not be very efficient and powerful, especially when the data is highly complex and non-linear. For some LDR methods, kernel tricks were proposed to improve classification performance [17-19]. The kernel trick aims to implicitly map the data that is not linearly separable onto higher dimensions hoping that the data become linearly separable or at least more "separable" than in the original space. Mapping implicitly is not feasible in all cases due to the complexity of kernelizing some LDR methods. Instead, the data could be explicitly mapped onto the target space and then LDR can be used on the mapped data.

In this paper, LDR combined with mapping the data onto higher dimensions is employed to classify precursor microRNAs from both pseudo hairpins and other non-coding RNAs. As discussed later, mapping the data onto higher dimensions can significantly improve the performance of the classifiers. In addition, using LDR can resolve the class imbalance problem since it takes the distribution of the data into consideration. As opposed to this, SVM only considers data near the margin. In addition, a feature selection method is proposed for selecting a subset of features instead of employing the whole feature vector, yielding very good results.

## Methods

### Datasets

The proposed classifier is able to distinguish human pre-microRNAs from both pseudo hairpins and other non-coding RNAs. The training dataset includes pre-microRNA sequences as the positive class and pseudo hairpins and other non-coding RNA sequences as the negative class. This dataset is available as stated in [13] as *supplementary material*. Detailed information about the datasets is given below.

#### Positive dataset

*Known human pre-microRNAs*: This dataset includes 691 non-redundant human pre-microRNA sequences, which are obtained from http://microrna.sanger.ac.uk/sequences/[20,21]. At first, 695 sequences were downloaded from miRBase and then after removing redundant sequences, 691 sequences were obtained which fold into hairpin structures. Some of these sequences fold into multi-branched loops at default parameters, showing sequences with multi-branched secondary structures that can also be identified by our classifier. We do not make any assumption about pre-microRNA secondary structures.

#### Negative dataset

*Pseudo hairpins*: The negative dataset is composed of 8,494 human pseudo hairpin sequences which were previously used in Triplet-SVM, MiPred, miPred and microPred. These sequences were obtained originally from RefSeq genes [22].

*Other non-coding RNAs*: This dataset contains all the non-coding RNA sequences, except microRNA sequences to make the classifier able to distinguish microRNA from other kinds of RNAs. This dataset contains 754 non-redundant sequences which are no longer than 150 nt. This dataset is known to be the best available ncRNA dataset for the human genome according to the authors of [23]. It includes 334 snoRNAs, 327 tRNAs, 53 snRNAs, 32 YRNAs, 5 5S-rRNAs, and three more sequences from other types of RNAs.

### Linear dimensionality reduction

Linear dimensionality reduction (LDR) is a well-known technique in pattern recognition. The basic idea of LDR is to represent an object of dimension *n *as a lower-dimensional vector of dimension *d*, achieving this by performing a linear transformation. The advantage of using a linear transformation is that, although the derivation of the underlying transformation may be slower, the classification is extremely fast as it performs linear-time operations to reduce to dimensions, typically, much lower than the original one.

We consider two classes, *ω*_1 _and *ω*_2_, which correspond to human pre-microRNAs as the positive class and pseudo hairpins and other ncRNAs as the negative class. The classes are represented by two normally distributed random vectors **x**_1 _*~ N *(**m**_1_, **S**_1_) and **x**_2 _*~ N *(**m**_2_, **S**_2_), respectively, with *p*_1 _and *p*_2 _the *a priori *probabilities. After the LDR is applied, two new random vectors **y**_1 _= **Ax**_1 _and **y**_2 _= **Ax**_2_, where **y**_1 _*~ N *(**Am**_1_; **AS**_1_**A***^t^*) and **y**_2 _*~ N *(**Am**_2_; **AS**_2_**A***^t^*) with **m***_i _*and **S***_i _*being the mean vectors and covariance matrices in the original space, respectively. The aim of LDR is to find a linear transformation matrix **A **in such a way that the new representations of the classes (**y***_i _*= **Ax***_i_*) are as separable as possible. Various criteria have been proposed to measure this separability [16]. We consider three LDR criteria: the well-known Fisher's discriminant analysis (FDA) [24,25], the heteroscedastic discriminant analysis (HDA) approach [26], and the Chernoff discriminant analysis (CDA) approach [16]. A brief discussion of these three follows.

The well-known FDA criterion consists of maximizing the Mahalanobis distance between the transformed distributions by finding a *d × n *matrix **A **that maximizes the following function [24]:

(1) $$J_{FDA}\left(A\right)=tr\left\{{\left(AS_{W}A^{t}\right)}^{-\text{1}}\left(AS_{E}A^{t}\right)\right\},$$

where **S***_W _*= *p*_1_**S**_1 _+*p*_2_**S**_2 _and **S***_E _*= (**m**_1 _- **m**_2_)(**m**_1 _- **m**_2_)*t *are the within-class and between-class scatter matrices respectively. The matrix **A **that maximizes (1) is obtained by finding the eigenvalue decomposition of:

(2) $$S_{FDA}\;=\;S_{W}^{-1}S_{E},$$

and taking the *d *eigenvectors whose eigenvalues are the largest ones. The resulting *d *eigenvectors are the rows of **A**.

HDA aims to obtain the matrix **A **that maximizes the function:

(3) $$J_{HDA}\left(A\right)\;=\;tr\left\{{\left(AS_{W}A^{t}\right)}^{-\text{1}}\left[AS_{E}A^{t}-{AS}_{W}^{\frac{1}{2}}\frac{p_{1}\text{log}\left({\text{S}}_{W}^{-\frac{1}{2}}{\text{S}}_{\text{1}}{\text{S}}_{W}^{-\frac{1}{2}}\right)+p_{2}\text{log}\left({\text{S}}_{W}^{-\frac{1}{2}}{\text{S}}_{\text{2}}{\text{S}}_{W}^{-\frac{1}{2}}\right)}{p_{1}p_{2}}{\text{S}}_{W}^{\frac{1}{2}}A^{t}\right]\right\}.$$

The solution to this criterion is given by computing the eigenvalue decomposition of:

(4) $$S_{HDA}=\;S_{{}_{W}}^{-1}\left[S_{E}-S_{W}^{\frac{1}{2}}\frac{p_{1}\text{log}\left({\text{S}}_{W}^{-\frac{1}{2}}{\text{S}}_{\text{1}}{\text{S}}_{W}^{-\frac{1}{2}}\right)+p_{2}\text{log}\left({\text{S}}_{W}^{-\frac{1}{2}}{\text{S}}_{\text{2}}{\text{S}}_{W}^{-\frac{1}{2}}\right)}{p_{1}p_{2}}{\text{S}}_{W}^{\frac{1}{2}}\right],$$

and choosing the *d *eigenvectors whose corresponding eigenvalues are the largest ones.

CDA aims to maximize the separability of the distributions in the transformed space, measured by the Chernoff distance between the two classes. CDA assumes that the classes are normally distributed (in the original and transformed spaces), maximizing the following function [16]:

(5) $$J_{CDA}\left(A\right)\;=\;tr\left\{p_{\text{1}}p_{\text{2}}AS_{E}A^{t}{\left(AS_{W}A^{t}\right)}^{-\text{1}}+\text{log}\left(AS_{W}A^{t}\right)-p_{1}\text{log}\left(AS_{1}A^{t}\right)-p_{2}\text{log}\left(AS_{2}A^{t}\right)\right\}.$$

In [16], a gradient-based algorithm was proposed, which maximizes the function of (5) iteratively. LDR is applied to each input vector and classification is performed via a Bayesian classifier.

### Non-linear mapping of linear dimensionality reduction

While LDR methods have been widely used in machine learning and pattern recognition due to their simplicity, there are some drawbacks in using linear transformations, especially when the data are non-linear and complex. Linear classifiers are usually inefficient compared to more sophisticated classifiers such as SVM.

Kernels have been used extensively in pattern recognition methods such as SVM, PCA and others. Also, it has been shown that FDA using kernels significantly improves the performance of LDR [17-19]. The main idea of kernel-based methods is to implicitly map input data to a higher dimension hoping the data become linearly separable. For some methods, the kernel trick allows one to solve the problem of mapping and classifying without explicitly mapping data to a higher dimensional space. However, this approach is not possible for LDR methods such as CDA and HDA for which an implicit solution is far from trivial.

On the other hand, explicit mapping is a good alternative in some scenarios. The problem that we work on is that the data are represented in a few dimensions (features), and a large number of samples. In fact, if implicit mapping solutions were available, the size of the kernel matrices would be in the order of the number of samples. In our dataset, it would be around 10,000 × 10,000 in which basic algebraic operations on the matrices are time and space consuming.

Another advantage for using LDR methods is also related to dealing with a large number of samples in a lower dimensional space. Even LDR methods are usually affected by the problem of singular matrices, it is not the case in our datasets. As a result of doing feature selection, we deal with a very small number of features, and even after mapping to a higher dimensional space, the number of new features does not increase to more than a hundred (depending on the choice of parameters), which is still very low compared to the number of samples (a few thousand).

Therefore, we propose to explicitly map the data onto a higher dimensional space and then apply LDR methods on the mapped data. Explicitly mapping the data can be a challenging task, since finding the actual mapping function *ϕ*(*x*) of kernels can be far from trivial, especially for the radial basis function (RBF) kernel. This is because RBF implicitly maps the data to an infinite dimensional Hilbert space. In this study, mapping functions that are extracted from polynomial and RBF kernels are used.

The authors of [27] employed linear SVM on polynomially mapped data in which the dataset is mapped explicitly. They applied the method on a large scale where the testing and training processes were very fast compared to polynomial kernels. The polynomial kernel has the following form:

(6) $$K\left(x_{i},x_{j}\right)\;=\;\;{\left(\gamma x_{i}^{T}x_{j}\;+\;r\right)}^{d}$$

where *γ *and *r *are the parameters, *d *is the degree of the polynomial and $x\in {\mathbb{R}}^{n}$. The product of two mapping functions *ϕ*(**x***_i_*) and *ϕ*(**x***_j _*) is the polynomial kernel. By setting *d *= 2, *r *= 1 and *γ *= 1 and simplifying the result, the mapping function results in:

(7) $$\varphi \left(x\right)\;=\;\;{\left[\text{1},\;x_{\text{1}},\;.\;.\;.\;,\;x_{n},\;x_{1}^{2}\;,\;.\;.\;.\;,\;x_{n}^{2},\;x_{\text{1}}x_{\text{2}},\;.\;.\;.\;,\;x_{n-1}x_{n}\right]}^{t}.$$

where *ϕ*(**x**) is of dimension *C*(*n *+ *d, d*). For explicit mapping, we need to use the simplified function to map the data and apply the LDR method on the new space.

As mentioned earlier, finding the mapping function of RBF is not a straightforward task. Rahimi and Recht [28] proposed a mapping mechanism that maps input samples to a randomized feature space with low dimensions, in which the inner product of any two mapped data is equivalent to a Gaussian RBF kernel of the two input data. Scikit-learn provides a package written in Python for approximating radial basis function kernel [29]. The package takes two parameters, *m *which is number of dimensions of the transformed features, and *gamma*, which is the parameter of the RBF. We use their package in our implementation.

### The features

One of the most important aspects of designing a classifier is to extract the most relevant features which empower the classifier to distinguish between classes of data. Therefore, extracting the set of appropriate features from the dataset is very momentous. In addition, since the datasets used in the training and testing processes contain multi-branched sequences, the extracted features should contain relevant information that can make the classifier able to succeed.

In our study, 48 features are used in which 29 of them were previously introduced in miPred and 19 features were proposed in microPred. While we provide here a brief description about the features, full details can be found in the supplementary material section of microPred [13]. These features can be categorized into two groups: thermodynamical and base pair-related features. *miPred *introduced 17 composition features including 16 dinucleotide frequencies and |*g *+ *c*|% ratio, 6 folding measure features, including modified base pairing propensity *dP*, modified minimum free energy (MFE) *dG*, modified base paire distance *dD*, modified Shannon entropy *dQ*, and MFE indices 1 and 2 (*MFEI*_1 _and *MFEI*_2_, respectively).

In addition, *microPred *introduced another 19 features including MFE index 3 and 4 (*MFEI*_3 _and *MFEI*_4_), normalized ensemble free energy (NEFE), MFE structure frequency (Freq), structural diversity (Diversity) and *Diff *= |*MFE *- *EFE*|/*L*, where EFE is the ensemble free energy and *L *is the length of the sequence. Also, some thermodynamical features were introduced, such as structure entropy *dS *and *dS/L*, structure enthalpy *dH *and *dH/L*, melting energy of the structure *Tm *and *Tm/L*. Additionally, a few base-pair-related features such as |*A *- *U*|/*L*, |*G *- *C*|/*L*,|*G *- *U*|/*L*, average base pairs per stem, where a stem is a structural motif in the secondary structure, and also %*|A - U|/nstems*, %|*G *- *C*|/*nstems*, %|*G *- *U*|/*nstems*, where *nstem *is number of stems in the secondary structure. All these features can be calculated and extracted from sequences of pre-microRNAs.

### The class imbalance problem

The class imbalance problem occurs when there is a large difference in prior class probabilities or when the disproportionate numbers of samples in positive and negative classes lead to poor performance of the classifier with respect to the smallest class [30]. As mentioned earlier, the number of positive samples in comparison to the negative samples is small, with a ratio of about 1:13. In this case, standard classifiers such as SVMs have tendency to classify well the largest class, while ignoring the smallest class.

In classifiers in which the numbers of samples in different classes are unbalanced, the performance of the classifier cannot be assessed accurately based on the percentage of test samples that are correctly classified. This is because, for example, when the number of samples in the negative class heavily outnumber the samples from the positive class and the classifier always classifies samples as negative, the accuracy is high, although the classifier is useless. Hence, other indicators are required for analysis of the classification performance. In [30], it is suggested that the *geometric mean*, $G_{m}\;=\;\sqrt{SE\;\times \;SP},$ can be a good indicator, where sensitivity (SE) and specificity (SP) are defined as follows:

(8) $$SP=\frac{TN}{TN+FP},$$

(9) $$SE=\frac{TN}{TP+FN},$$

Here, *TP *represents pre-microRNAs which are correctly classified as pre-microRNA (true positives), *TN *represents non pre-microRNAs (pseudo hairpins and other ncRNAs) which are correctly classified as non pre-microRNA (true negatives), *FP *represents non pre-microRNAs which are misclassified as pre-microRNA (false positives) and *FN *represents pre-microRNAs which are incorrectly classified as non pre-microRNA (false negatives). Using LDR as the classifier is a good strategy to overcome the class imbalance problem. This is because LDR methods take the distribution of the whole data into account, in order to optimize the prediction function. This is in contrast with the criterion followed by the SVM that uses only the "support vectors" to find the most efficient prediction function. Although some SVM schemes have improved this by incorporating the concept of soft margin, the SVM still relies on the vectors on (or next to) the margin, ignoring the contribution of the other samples to a more efficient classification rule. The latter feature is, indeed, intrinsic to LDR techniques.

### Feature selection

Feature selection is important for a variety of reasons: increasing the generalization power, speeding up the training and testing processes, improving classification performance, and result comprehensibility [31]. Feature selection algorithms can be widely categorized into two groups: filter and wrapper methods. Filter methods evaluate the "goodness" of the feature subset by using the intrinsic characteristics of the data. They are computationally cheap, since they do not involve the induction algorithm. However, they also take the risk of selecting subsets of features, which may not match the chosen induction algorithm. Wrapper methods, on the contrary, directly use the induction algorithm to evaluate the feature subsets. They generally outperform filter methods in terms of prediction accuracy, but are computationally more intensive [32]. Brute-force search is a method that evaluates the performance of the classifier based on different subsets of features. In this method, the performance of all possible subsets of features are compared to each other. In other words, the performance of all possible two-feature pairs are compared to the performance of all possible subsets of three features and so on. Despite the fact that brute-force search guarantees the highest accuracy, it is extremely time-consuming and impractical - brute-force search should find the best subset of features among the 2*^n ^*possible subsets, where *n *is the number of features. Thus, the search space is extremely large that it is not possible to run this method for more than a few features. Another feature selection method is forward search which is a greedy algorithm to find a sub-optimal subset of features [33]. This algorithm starts with the null set and selects features to be added to the set one at a time, based on the performance of the classifier with the currently selected features in addition to a potential selected feature. This algorithm is very fast and usually has an acceptable performance, but does not guarantee the best subset of features.

In this study, we introduce a systematic feature selection method that is based on floating forward search feature selection and aims to improve the performance of the basic algorithm. The improvement relies on searching a larger feature space compared to the basic forward search approach. In our approach, the best 10 pairs of features among all the pairs (2-tuples) of features are selected. Then, all combinations of pairs with a third feature are evaluated and the best 10 triplets (3-tuples) of features are selected. This procedure is continued with *k*-tuples, *k *= 4, 5*, . . *. until a criterion is satisfied. The criterion can be a certain number of features being selected or selecting a new feature that does not improve the performance significantly. In our approach, the feature selection process is continued until 12 features are evaluated.

As mentioned earlier, since our dataset is unbalanced, *G_m _*is used to measure the performance of the classifiers to ensure that class imbalance does not mislead feature selection algorithms to select the best subset of features, regardless of different proportions between the number of samples in each class. Combining feature selection with explicit mapping followed by LDR and Bayesian classification yields the prediction model proposed in this paper - we call it miLDR-EM.

## Results and discussion

In this study different experiments have been conducted on the dataset described in the Methods section. In order to evaluate the performance of the classifiers and feature selection mechanisms, 10-fold cross validation is used. The partitioning is done in such a way that the ratio between the number of samples in the positive class and the number of samples in the negative class remain the same. At each stage, one of these groups is selected as the test set and the other nine subsets are used as the training set. In this method, no sample from the test set is used in the training process and only the test set can be used for evaluating the performance of the classifier.

In the first experiment, the mapping is applied to the dataset with pairs of features, using the polynomial and RBF mapping functions. Then, it is compared to the performance of the miLDR-EM classifier with the best subset of features in the dataset. Table 1 shows a comparison between LDR-based classifiers with different methods for explicit data mapping. The first row shows the results of LDR without any mapping at all. As mentioned in the Methods section, there are different combinations of LDR criteria (FDA, HDA and CDA) and two classification mechanisms (linear and quadratic). Here, only the best results of these six combinations are shown in Table 1. As we can see, the classifier that achieved the best performance when the data consists of explicitly mapping onto higher dimensions using the RBF mapping function employing HDA coupled with a quadratic classifier.

**Table 1:** Comparison of different mapping functions for LDR-based methods.

Mapping Method	SE	SP	*G_m_*
No mapping	69.17%	98.94%	82.72%
Polynomial	76.99%	96.98%	86.41%
RBF	85.23%	92.90%	88.99%

A comparison between mapping functions, polynomial and RBF, when using LDR on different pairs of features.

In order to evaluate the feature selection method, a comparison between the performance of the proposed system using the whole dataset versus a subset of features was performed. The aim is to select a subset of features and measure the goodness of that subset using a particular classifier. If mapping the data is required, all the samples with the selected features will be mapped to the target high dimensional space. Then, using 10-fold cross validation, the data is partitioned into ten subsets and the LDR-based classifier will evaluate the performance of the system. At this stage, all the intermediate results are stored in a database to be used for next round of feature selection.

**Figure 1:**
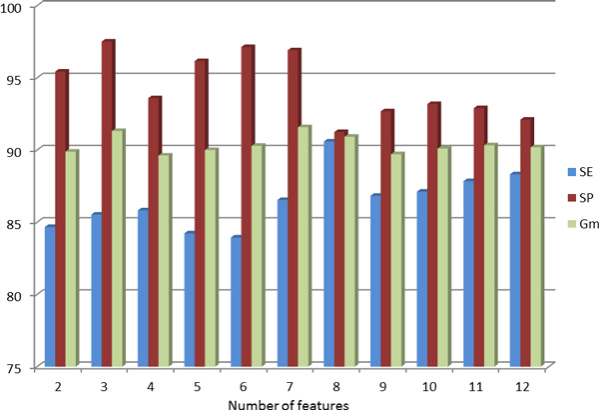
**Performance for different subsets of features**. Performance of miLDR-EM when using different subsets of features with RBF mapping. The *x*-axis represents the size of the feature subset, while the *y*-axis corresponds to the performance values for the three measures, SE, SP and *G_m_*, achieved by miLDR-EM with RBF mapping.

Table 2 compares the results of the feature selection method with explicitly mapped data for different numbers of features. The mapping function that has been used in this experiment is RBF with default parameters, *γ *= 1.5 and *m *= *n *+ 15, where *m *is the number of dimensions we map the data and *n *is the number of features in the feature subset. The classifier performs very well with just three features yielding *G_m _*= 91.32%. Also, it achieves *G_m _*= 91.58% with seven features. Figure 1 depicts a plot of the performance values for SE, SP and *G_m_*, and helps clearly visualize the trends of these measures for different numbers of features. The plot shows how the performance, measured by *G_m_*, shows two peaks at 3 and 7, and stays steady for other numbers of features.

**Table 2:** Results for feature selection.

No. of Features	SE	SP	*Gm*
2	84.67%	95.43%	89.89%
3	85.53%	97.51%	91.32%
4	85.82%	93.59%	89.62%
5	84.23%	96.16%	90.00%
6	83.94%	97.13%	90.29%
7	86.54%	96.91%	91.58%
8	90.59%	91.24%	90.92%
9	86.83%	92.68%	89.71%
10	87.12%	93.18%	90.10%
11	87.84%	92.90%	90.33%
12	88.32%	92.10%	90.19%

Performance of feature selection with miLDR-EM for different numbers of features. The three measures of performance are SE, SP and *G_m_*.

**Figure 2:**
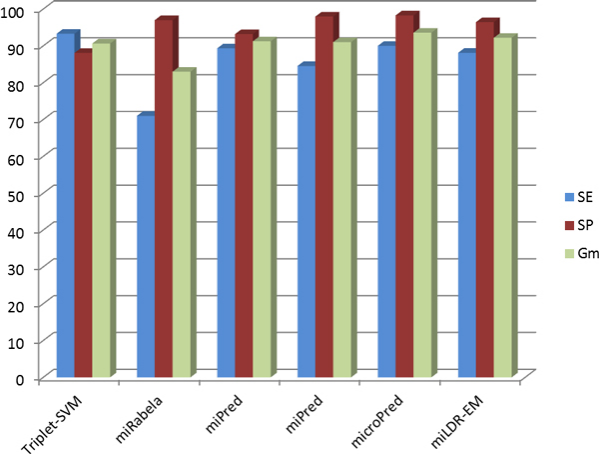
**Results for different methods**. Performance comparison of previously proposed methods against miLDR-EM. The *x*-axis represents each method for the three different measures, SE, SP and *G_m_*, while the *y*-axis corresponds to the values achieved for the three measures.

**Figure 3:**
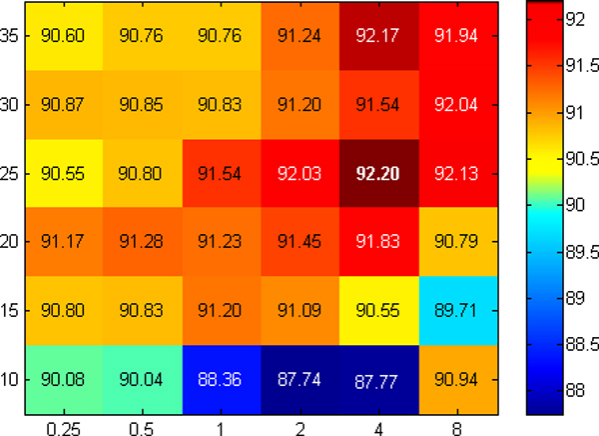
**Plot of parameter optimization for three features**. Heatmap containing *G_m _*values for different values used for *γ *(*x*-axis) and *nComponents *(*y*-axis) in the parameter optimization phase for three features. The colors range from dark blue (lowest value) to dark red (largest values). The largest value, *G_m _*= 92.20, is highlighted in bold. Some values are shown in white just to enhance visualization.

**Figure 4:**
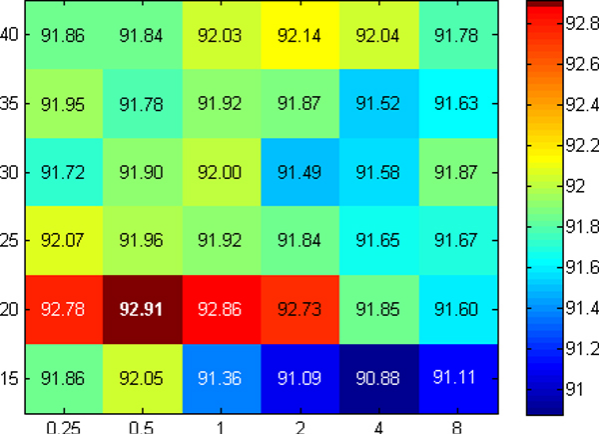
**Plot of parameter optimization for seven features**. Plot for the parameter optimization phase for seven features. The heatmap plots the values of *Gm *for different values of *γ *(*x*-axis) and *nComponents *(*y*-axis). In bold is the largest value of *G_m_*. Some numbers are shown in white just to enhance visualization.

Furthermore, in order to optimize the parameters of the RBF mapping, a grid search was performed. The values that were used for *γ *are [0.25, 0.5, 1, 2, 4, 8], and for *nComponents *are [10,15,20,25,30] for three features and [15, 20, 25, 30, 35] for seven features. Figures 3 and 4 show heatmaps containing *G_m _*values for all possible values of *γ *and *nComponents*, for three and seven features respectively. For three features, the values of *G_m _*are spread on a larger range from 87.74 to 92.20 (the maximum). Although there are small values in the plots (most of them for *nComponents *= 10), most of the values are reasonable large, with quite a few of them near the maximum. This demonstrates the flexibility of miLDR-EM for a wide range of parameters. A similar case is also observed for seven features. The heatmap looks different because the color map is on a smaller range. The heatmap has been plotted in this way in order to visualize the largest values (*G_m _≥ *92.73) in red, and make the distinction with the smaller values, which otherwise will not be too different from larger ones. As in three features, the smaller values are for *nComponents *= 15. Also, there are many cases with large values, again, showing the flexibility of miLDR-EM for a wide range of values for *nComponents*. Overall, miLDR-EM is more flexible for seven than for three features. This is reasonable as using only three features involves a much smaller number of possible subsets, whereas for seven features the number of possible subsets is much larger. Although the feature selection mechanism does not exhaustively search all possible subsets for seven features, it selects those among the best ones for classification, yielding more chances of obtaining higher classification performance, even by widening the range of parameter values.

In Table 3, the optimized parameters and corresponding performances are shown. As it is clear from the table, with three features, the classifier achieved *G_m _*= 92.20% and with seven features *G_m _*= 92.91%. The standard deviations for both cases are very low, considering that the cross validation is ten-fold and the number of features is small. The performance of the classifier with three features is very good, considering that the classifier uses a very small number of features. As mentioned in the Methods section, using fewer features reduces the complexity of the system and gives an opportunity for the researchers to understand the relation between the features from a biological perspective. Therefore, it is remarkable that although seven features yields slightly better performance, using three features for classification is justifiable with *G_m _*= 92.20%.

**Table 3:** Results for optimized parameters.

No. of Features	Gamma	*m*	SE	SP	*G_m_*	**Std**.
3	4.00	25	88.13%	96.45%	92.20%	±0.068
7	0.50	20	89.15%	96.84%	92.91%	±0.056

Optimized parameters for RBF mapping when using *three *and *seven *features. The last column represents the standard deviation of the *G_m _*values for the ten folds.

### Comparison with other methods

We compared LDR coupled with RBF mapping, which yields the highest performance when using three features, with some of the previously proposed methods as follows: miRabela [8], miPred [12], MiPred [11], microPred [13], and Triplet-SVM [9]. The result of the comparison is presented in Table 4. The plot of the performance values are more clearly visualized in Figure 2, in which the *x*-axis corresponds to the methods, and the bars represent the three measures of performance, SE, SP and *G_m_*.

**Table 4:** Comparison with other methods.

Method	**Ref**.	**Feat**.	SE	SP	*G_m_*
Triplet-SVM	[9]	32	93.30%	88.10%	90.66%
miRabela	[8]	40	71.00%	97.00%	82.99%
miPred	[12]	34	89.35%	93.21%	91.26%
miPred	[12]	29	84.55%	97.97%	91.01%
microPred	[13]	21	90.02%	98.28%	93.58%
miLDR-EM	-	3	88.13%	96.45%	92.20%
miLDR-EM	-	7	89.15%	96.84%	92.91%

Performance comparison of miLDR-EM with previously proposed methods. The first column contains the name of the method being compared, the second column contains the number of features being used by that method, and the subsequent columns represent SE, SP and *G_m _*respectively. For all previous methods, the results in the table are those reported in [13].

The results included in Table 4 correspond to those reported in [13], since that paper shows the geometric mean as a comparison, while the others focus on accuracy. For the reasons discussed earlier in this paper, classification accuracy is expected to be much higher than *G_m_*, at the expense of misclassifying most samples in the smallest class, if not all of them. Triplet-SVM used a complete dataset of 193 positive and 8,494 negative samples. Of these, several samples were taken randomly (without replacement) to create non-overlapping training and test sets, containing 163 positive and 168 negative, and 30 positive and 1,000 negative samples respectively. From these numbers, it is obvious that the classifier will not face the imbalance problem on the training data. However, randomly picking samples from the complete dataset could produce a biased classifier toward some particular distribution (as no cross-validation is used). The same datasets were also used for testing miPred and microPred, but with different numbers of features (as indicated in the table). In addition, as pointed out in [13], no clear details were given in [8] on how the training and test sets were obtained. The performance of that method is very low though, and it uses 40 features. microPred used the same dataset as that of our experiments, with a 5-fold cross-validation process and a separate dataset of randomly chosen samples for test purposes.

Although the performance of miLDR-EM is slightly lower than that of microPred, miLDR-EM uses only *three *features and performs very well compared to microPred, which uses 21 features and achieves a *G_m _*value of just 1.38% higher than miLDR-EM. Even better performance is achieved by miLDR-EM with *seven *features, 0.67% lower than that of microPred with 21 features. The other advantage that miLDR-EM shows with respect to other methods is that the difference between SE and SP is much smaller, showing the power of the proposed scheme for solving the class imbalance problem. Only miPred and Triplet-SVM achieve a better balance between SE and SP, but yield a smaller *G_m_*, and use a much larger subset of features, 34 and 32 respectively. Although microPred and miLDR-EM achieve a similar balance between SE and SP, miLDR-EM uses fewer features. In addition, the plot of Figure 1 reveals that selecting eight features does not lower the performance substantially and keeps an almost perfect balance between SE and SP.

Overall, due to the small difference between the two methods, we can assert that miLDR-EM is the best choice, since it can achieve comparable results with a much smaller subset of features, yields a smaller difference between SE and SP, and can also provide an additional biological insight (discussions at the end of this section). This may provide not only an improvement on the computational task for classification, but also a more meaningful insight on the RNA structural properties that are suitable for prediction of pre-microRNA and pseudo hairpins. Note that we have tested miLDR-EM with one and two features, and the performance lowers to a *G_m _*value under 90%. For this reason, selecting three features is the best choice.

To conclude this section, a discussion on the selected features is presented. The three features that miLDR-EM uses for classification are the following: *dG*, *zG *and *NEFE*. *dG *represents the normalized free energy of folding in the sequences, normalized by the length of the sequence. *zG *is the normalized variant (*z*-score) for feature *dG*. *NEFE *measures the normalized ensemble free energy of the sequence. As *dG*, *NEFE *is also normalized by the length of the sequence. In a nutshell, it can be inferred that the identification of microRNA is merely based on minimum free energy (and its normalized *z*-score), and the normalized ensemble free energy. The other 45 features are much less relevant or not relevant at all in the prediction problem, and could eventually be disregarded.

## Conclusion

We have proposed a classification approach for identifying microRNA sequences and classifying pre-microRNAs from pseudo hairpins and other non-coding RNAs, which we call miLDR-EM. We have used linear dimensionality reduction (LDR) as the main classification engine. The proposed approach explicitly maps the data onto a higher dimensional space and classification takes place on the mapped data. While using different functions for mapping the data, the results show that RBF helps the classifier perform better. Also, a feature selection method is applied on the dataset in order to find a subset of relevant features. The results turn out to be very good compared to previously proposed methods. With only three features, miLDR-EM performs slightly lower than microPred which uses 21 features. Explicitly mapping the data onto a higher dimensional shows significant improvement in performance. This study shows that in cases in which the number of samples is very large, explicit mapping can be used instead of kernel-based methods. In addition, we propose a specialized feature selection method in which we keep a balance between exhaustive search and greedy search for finding a significantly smaller subset of features.

## Competing interests

The authors declare that they have no competing interests.

## Authors' contributions

Both authors contributed equally to this work.

## Declarations

The publication costs for this article were funded by the Natural Sciences and Engineering Council of Canada, NSERC.

This article has been published as part of *BMC Proceedings *Volume 7 Supplement 7, 2013: Proceedings of the Great Lakes Bioinformatics Conference 2013. The full contents of the supplement are available online at http://www.biomedcentral.com/bmcproc/supplements/7/S7.
